# Recurrent Stroke Due to Pulmonary Arteriovenous Malformation: Diagnostic Challenges and Management

**DOI:** 10.7759/cureus.98393

**Published:** 2025-12-03

**Authors:** Rui Pedro Ribeiro, Carolina Guimarães, Helena Hipólito Reis, Ana Aires, Ana Pastor, Luísa Fonseca

**Affiliations:** 1 Department of Internal Medicine, Unidade Local de Saúde de São João, Porto, PRT; 2 Department of Neurology, Unidade Local de Saúde de São João, Porto, PRT

**Keywords:** cryptogenic stroke, endovascular interventions, paradoxical embolism, pulmonary arteriovenous malformation, right-to-left shunt

## Abstract

Pulmonary arteriovenous malformations (PAVMs) are rare vascular anomalies that establish a direct right-to-left shunt (RLS). Although uncommon, they may cause paradoxical embolism leading to stroke, cryptogenic stroke, or hemorrhage. Despite their potential severity, PAVMs remain an unrecognized entity.

We report a case of a 57-year-old woman with cardiovascular risk factors and a previous recurrent stroke of undetermined origin, admitted with acute right-sided weakness, dysarthria, and facial droop. Computed tomography (CT) confirmed acute infarcts in the left frontal and temporal cortices. During the diagnostic workup, transcranial Doppler with bubble contrast revealed RLS, and transesophageal echocardiography excluded an intracardiac defect. Pulmonary CT angiography identified a right lower lobe PAVM that was embolized with an Amplatzer Vascular Plug 4 (St. Paul, MN: Abbott). Further investigations excluded other etiologies. A comprehensive investigation of RLS is crucial when no other etiology for a stroke is found. Transcranial Doppler and CT angiography are key tools for diagnosis, and embolization is an effective treatment.

## Introduction

Pulmonary arteriovenous malformation (PAVM) is characterized by the presence of abnormal pulmonary blood vessels in which there is a direct connection between arterial and venous vessels. This bypass of the capillary bed can create a low-resistance and high-flow continuous intrapulmonary right-to-left shunts (RLS), which can lead to a broad spectrum of clinical manifestations, including life-threatening hemorrhage or complications from paradoxical embolization (e.g., ischemic stroke or brain abscess) [1,2]. In patients with no clear cardioembolic source but with evidence of an RLS, paradoxical embolism should be considered. While a patent foramen ovale is a common cause, PAVMs remain an underrecognized entity, with retrospective studies showing an incidence of PAVMs in approximately 0.02% of patients with ischemic stroke [3]. Early diagnosis and management are essential to prevent recurrent cerebral ischemic events. This case emphasizes the relevance of investigating pulmonary sources of embolism when standard stroke workup is unrevealing.

## Case presentation

A 57-year-old right-handed woman presented to the emergency department two hours after the sudden onset of right-sided limb weakness, trouble speaking, and facial droop. She was a smoker (more than 20 cigarettes daily for over 40 years), and had a history of arterial hypertension, dyslipidemia, previous hepatitis C virus infection, and a past of cocaine and heroin use. She had experienced several strokes in multiple territories of undetermined etiology (at that date, transthoracic echocardiogram, 24 h ambulatory electrocardiography, and transcranial Doppler ultrasound had revealed no obvious abnormality) and, since then, had been on antiplatelet therapy.

A physical examination revealed normal vital signs and glycemia, but weakness in her right upper and lower limbs (graded 4/5 according to the Medical Research Council scale), partial right facial paralysis, and severe dysarthria. Her National Institutes of Health Stroke Scale (NIHSS) score was 6, and her modified Rankin Scale (mRS) was 1. A non-contrast head computed tomography scan revealed no acute lesions, and the patient was infused with a standard dose of intravenous recombinant tissue plasminogen activator.

Subsequent vascular studies were performed and excluded the presence of luminal filling abnormalities on carotid and vertebral vessels. Brain magnetic resonance imaging revealed three ischemic acute lesions in the cortical areas of the left frontal and temporal regions. The chest X-ray showed no abnormal cardiopulmonary findings.

**Figure 1:**
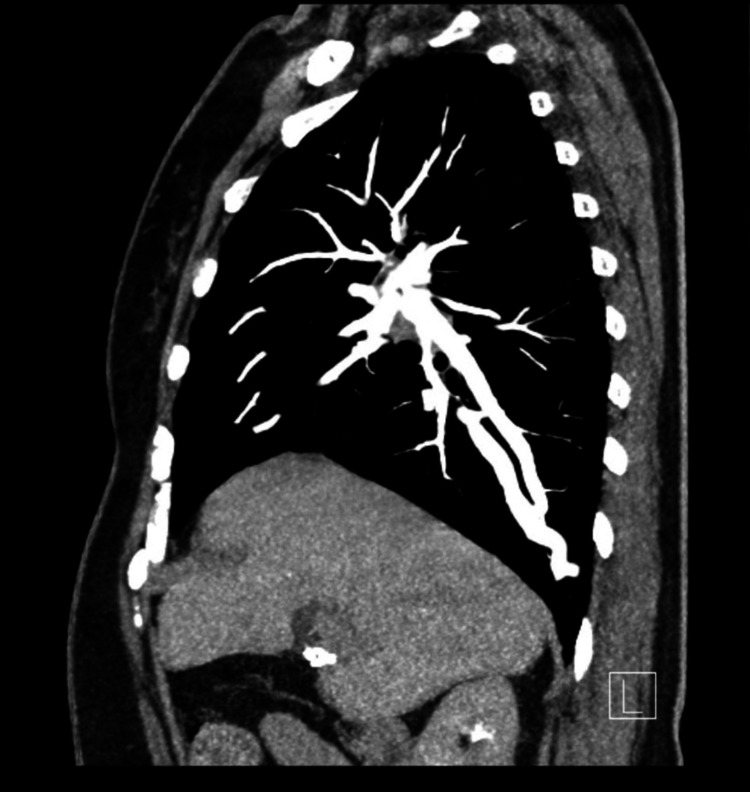
CT angiography revealed a right lower lobe PAVM. PAVM: pulmonary arteriovenous malformations

As part of the etiologic workup, transcranial Doppler with bubble study found significant right-to-left shunting, classified as severe (grade 4/4). Transesophageal echocardiography excluded an intracardiac shunt; however, microbubbles were observed entering the left atrium via the pulmonary veins, raising suspicion of an intrapulmonary shunt. Pulmonary CT angiography revealed a right lower lobe PAVM measuring 24×18 mm (transverse×anteroposterior) as follows: the feeding artery, originating from the right pulmonary artery, had a maximum upstream diameter of 6 mm, while the draining pulmonary vein measured 7 mm (Figure 1).

**Figure 2:**
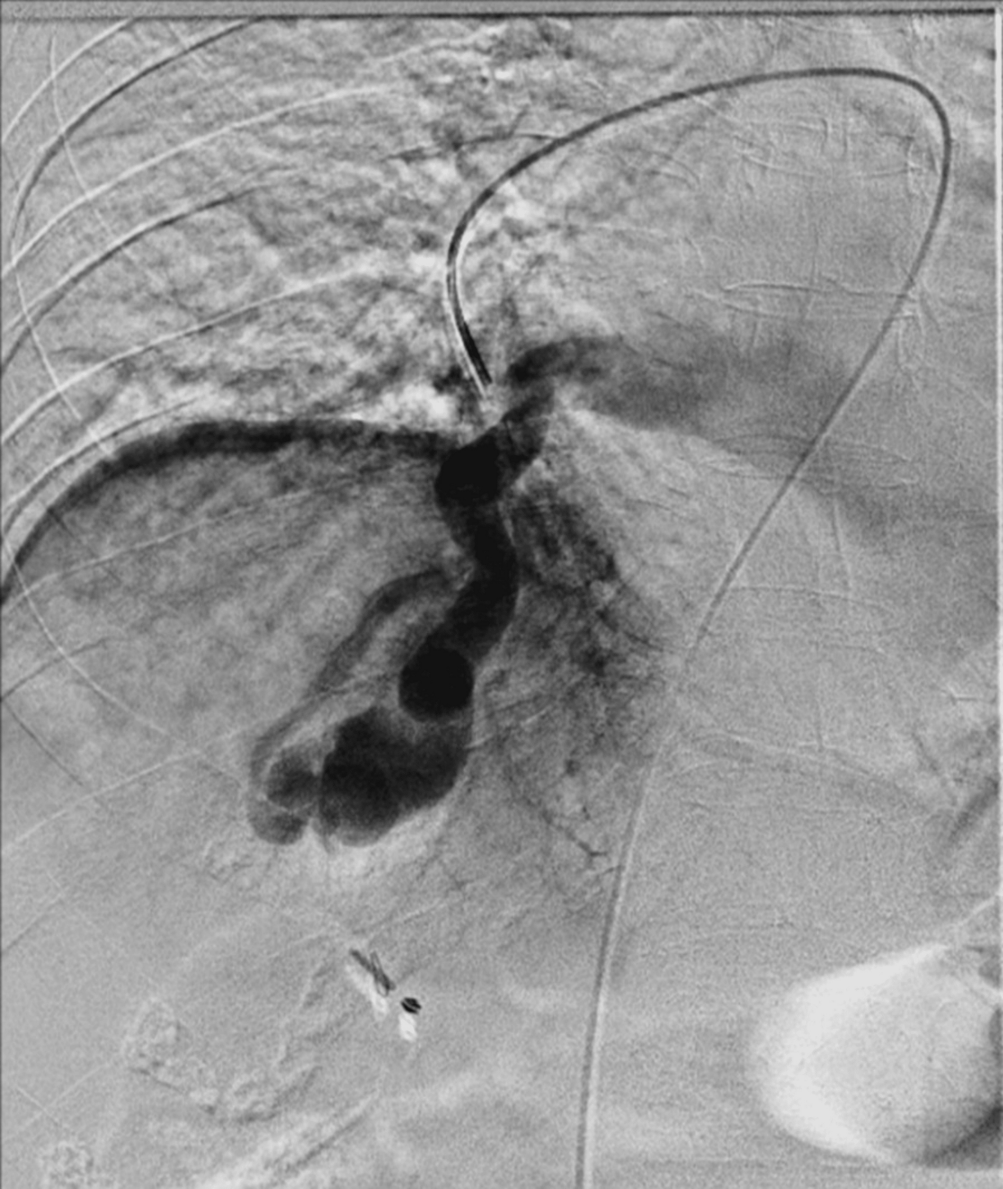
Pulmonary angiography confirmed a high-flow PAVM. PAVM: pulmonary arteriovenous malformations

Pulmonary angiography confirmed a high-flow PAVM. Superselective catheterization of the right lower lobar pulmonary artery and basal segmental branch demonstrated a feeding artery with a distal diameter of 5 mm and a mid-segment diameter of 6 mm with no other vascular malformations identified (Figure 2).

**Figure 3:**
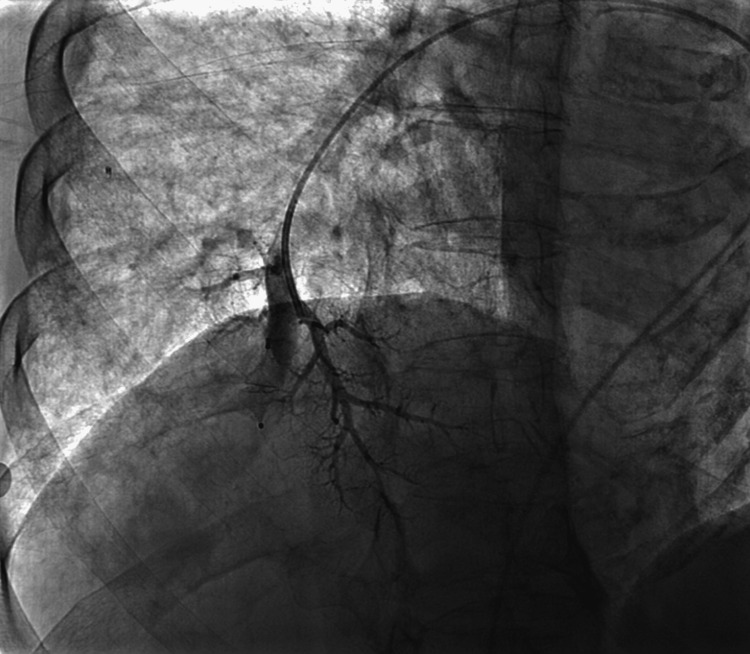
Control angiography confirmed successful endovascular exclusion of the PAVM. PAVM: pulmonary arteriovenous malformations

Selective catheterization of the right pulmonary artery and the feeding artery of the right posterior basal PAVM was performed. An Amplatzer Vascular Plug 4 (St. Paul, MN: Abbott) (8×13.5 mm) was deployed, and control angiography confirmed successful endovascular exclusion of the PAVM (Figure 3).

Some causes of acquired malformations were excluded, namely cirrhosis (no parenchymal abnormalities on ultrasound and hepatic elastography of 4 kPa), pulmonary tuberculosis or other infections (CT scan showing no parenchymal disease or other abnormalities), and no prior history of trauma or surgery. Venous Doppler of the lower limbs was negative for deep vein thrombosis. Atrial fibrillation was not detected on telemetry during her hospital stay. Laboratory investigations ruled out hematological abnormalities or hypercoagulable states. Given the absence of a family history of hereditary hemorrhagic telangiectasia (HHT) and the lack of previous symptoms reported (such as nasal or other bleeding), HHT was considered unlikely, and genetic testing was not pursued.

The patient showed improvement in neurological deficits, with only slight residual weakness in the lower limb. She has maintained follow-up and underwent repeat CT angiography, which demonstrated persistent occlusion of the PAVM. To date, no new embolic events have occurred, and the patient remains on antiplatelet therapy.

## Discussion

This case demonstrates a rare but significant cause of paradoxical embolism due to a pulmonary arteriovenous malformation. Most cases are associated with HHT, but isolated lesions, as in this patient, may occur [4].

According to several studies, transcranial Doppler with bubble contrast has greater sensitivity for detecting RLS than echocardiography and should be the preferred screening method [5,6]. Transesophageal echocardiography is important to distinguish between intracardiac and intrapulmonary shunt [7]. The identification of microbubbles entering the left atrium through pulmonary veins strongly supports an intrapulmonary shunt. In patients with cryptogenic stroke, particularly those with recurrent events or involving multiple arterial territories, investigation for right-to-left shunt is critical.

When a PAVM is suspected, CT angiography is the modality of choice for its diagnosis, as it allows us to characterize the lesion, identify the feeding artery, and plan a possible therapeutic intervention. Although angiography is the gold standard, it should be used only in patients at the time of endovascular treatment [8].

Treatment of PAVMs, according to several studies, is indicated when feeding arteries exceed 3 mm, regardless of the presence or absence of symptoms. Endovascular embolization is the standard of care, with excellent efficacy in preventing further neurologic events [9-12]. According to some studies, the long-term success rate of embolization is over 80% [11].

Although approximately 80% of PAVMs are congenital and therefore associated with conditions, such as HHT, when this does not appear to be the most likely underlying cause, other etiologies should be excluded, including trauma, liver cirrhosis, infections (such as fungal infections or tuberculosis), or a history of previous surgery [13]. The absence of typical HHT signs (e.g., epistaxis, mucocutaneous telangiectasia, family history) supported a non-HHT etiology in this patient. With no other embolic sources identified, the likely mechanism of stroke was paradoxical embolism through the PAVM.

## Conclusions

This case highlights the importance of comprehensive evaluation of patients with cryptogenic strokes, especially when the initial investigation is inconclusive. Recognition and treatment of pulmonary shunts are crucial, as they require specific management and have significant prognostic value.

## References

[1] Faughnan ME, Granton JT, Young LH (2009). The pulmonary vascular complications of hereditary haemorrhagic telangiectasia. Eur Respir J.

[2] Post MC, van Gent MW, Plokker HW (2009). Pulmonary arteriovenous malformations associated with migraine with aura. Eur Respir J.

[3] Topiwala KK, Patel SD, Pervez M, Shovlin CL, Alberts MJ (2021). Ischemic stroke in patients with pulmonary arteriovenous fistulas. Stroke.

[4] Shovlin CL, Condliffe R, Donaldson JW, Kiely DG, Wort SJ (2017). British Thoracic Society clinical statement on pulmonary arteriovenous malformations. Thorax.

[5] Zhang D, Jiang L, Chen YN, Pan MF (2024). The diagnostic value of contrast-enhanced transcranial Doppler and contrast-enhanced transthoracic echocardiography for right to left shunt in patent foramen ovale: a systematic review and meta-analysis. Front Neurol.

[6] Tian L, Zhang M, Nie H, Zhang G, Luo X, Yuan H (2023). Contrast-enhanced transcranial Doppler versus contrast transthoracic echocardiography for right-to-left shunt diagnosis. J Clin Monit Comput.

[7] Katsanos AH, Psaltopoulou T, Sergentanis TN (2016). Transcranial Doppler versus transthoracic echocardiography for the detection of patent foramen ovale in patients with cryptogenic cerebral ischemia: a systematic review and diagnostic test accuracy meta-analysis. Ann Neurol.

[8] Kramdhari H, Valakkada J, Ayyappan A (2021). Diagnosis and endovascular management of pulmonary arteriovenous malformations. Br J Radiol.

[9] Faughnan ME, Palda VA, Garcia-Tsao G (2011). International guidelines for the diagnosis and management of hereditary haemorrhagic telangiectasia. J Med Genet.

[10] Gossage JR, Kanj G (1998). Pulmonary arteriovenous malformations. A state of the art review. Am J Respir Crit Care Med.

[11] Mager JJ, Overtoom TT, Blauw H, Lammers JW, Westermann CJ (2004). Embolotherapy of pulmonary arteriovenous malformations: long-term results in 112 patients. J Vasc Interv Radiol.

[12] Cottin V, Plauchu H, Bayle JY, Barthelet M, Revel D, Cordier JF (2004). Pulmonary arteriovenous malformations in patients with hereditary hemorrhagic telangiectasia. Am J Respir Crit Care Med.

[13] Zhan J, Dong C, Li M, Zhan L, Chen H, Lu L, Liu J (2021). Cryptogenic stroke caused by pulmonary arterial venous malformation with massive right-to-left shunt: a case report. Neurol Ther.

